# ShinyFruit: interactive fruit phenotyping software and its application in blackberry

**DOI:** 10.3389/fpls.2023.1182819

**Published:** 2023-10-05

**Authors:** T. Mason Chizk, Jackie A. Lee, John R. Clark, Margaret L. Worthington

**Affiliations:** Department of Horticulture, University of Arkansas, Fayetteville, AR, United States

**Keywords:** image analysis, postharvest quality, red cell regression, red drupelet reversion, reddening, *Rubus* subgenus *Rubus*

## Abstract

**Introduction:**

Horticultural plant breeding programs often demand large volumes of phenotypic data to capture visual variation in quality of harvested products. Increasing the throughput potential of phenomic pipelines enables breeders to consider data-hungry molecular breeding strategies such as genome-wide association studies and genomic selection.

**Methods:**

We present an R-based web application called ShinyFruit for image-based phenotyping of size, shape, and color-related qualities in fruits and vegetables. Here, we have demonstrated one potential application for ShinyFruit by comparing its estimates of fruit length, width, and red drupelet reversion (RDR) with ImageJ and analogous manual phenotyping techniques in a population of blackberry cultivars and breeding selections from the University of Arkansas System Division of Agriculture Fruit Breeding Program.

**Results:**

ShinyFruit results shared a strong positive correlation with manual measurements for blackberry length (*r* = 0.96) and ImageJ estimates of RDR (*r* = 0.96) and significant, albeit weaker, correlations with manual RDR estimation methods (*r* = 0.62 - 0.70). Neither phenotyping method detected genotypic differences in blackberry fruit width, suggesting that this trait is unlikely to be heritable in the population observed.

**Discussion:**

It is likely that implementing a treatment to promote RDR expression in future studies might strengthen the documented correlation between phenotyping methods by maximizing genotypic variance. Even so, our analysis has suggested that ShinyFruit provides a viable, open-source solution to efficient phenotyping of size and color in blackberry fruit. The ability for users to adjust analysis settings should also extend its utility to a wide range of fruits and vegetables.

## Introduction

The value of a horticultural product is almost always influenced by its appearance, which includes color, size, shape, and other morphological components. These characteristics are often important indices of maturity, harvest efficiency, structural integrity, disease, insect damage, and flavor. Even when flavor differences are not present, individual consumers perceive differences in flavor intensities between differently colored but otherwise identical, food products (Bayarri et al., 2001; Shankar et al., 2009). The United States Department of Agriculture, Agricultural Marketing Service (USDA-AMS) has implemented visual grading and inspection guidelines for nearly all fresh-market fruit and vegetable crops (United States Department of Agriculture, Agricultural Marketing Service, 2017). Inspectors assess color and shape using subjective visual techniques, which can become costly and time-consuming for researchers when a high degree of accuracy is desired (Abebe et al., 2023).

With modern photography and computing, it is possible to construct low-cost objective phenotyping pipelines that are high throughput and based exclusively in open-source software. ImageJ software is a general user interface (GUI) enabled tool that has been widely used to construct such phenotyping pipelines (Maloney et al., 2014; Cortes et al., 2017; Chizk, 2018). Unfortunately, using ImageJ for customized batch image processing requires an understanding of the ImageJ macro programming language, which is a java-based language somewhat restrictive in scope to the utilities present in the tool. PlantCV is a Python-based tool that presents an alternative to ImageJ, but its effective implementation requires some knowledge of the Python programming language (Gehan et al., 2017). Furthermore, as a generalized tool for plant image analysis, PlantCV contains a large number of functions and lengthy documentation. It has been used effectively in a number of studies for high-throughput image phenotyping (Enders et al., 2019; Kumar et al., 2020; Chang et al., 2021; Marrano and Moyers, 2022), but like ImageJ, this tool may present a steep learning curve to some users.

We present an alternative R-based approach (R Core Team, 2022) called ShinyFruit, which is a software package that offers an interactive GUI designed to simultaneously perform color, size, and shape analyses on large sets of fruit images. ShinyFruit users currently can detect fruit in.jpg images by setting color threshold values in red-green-blue (RGB), hue-saturation-brightness (HSB), and L*a*b* color spaces. Following fruit detection, the user can indicate a size reference and select from a list of traits to include in the.csv output file. ShinyFruit is primarily intended to be used by researchers who have an interest in measuring visual characteristics of fruit and vegetables for the purpose of breeding, estimating the incidence or severity of disease or insect pest injury, or investigating the effects of farm management practices on quality. Secondary users might include processors, distributors, or farmers that wish to evaluate samples of fruit at various stages of the supply chain.

**Figure 1:**
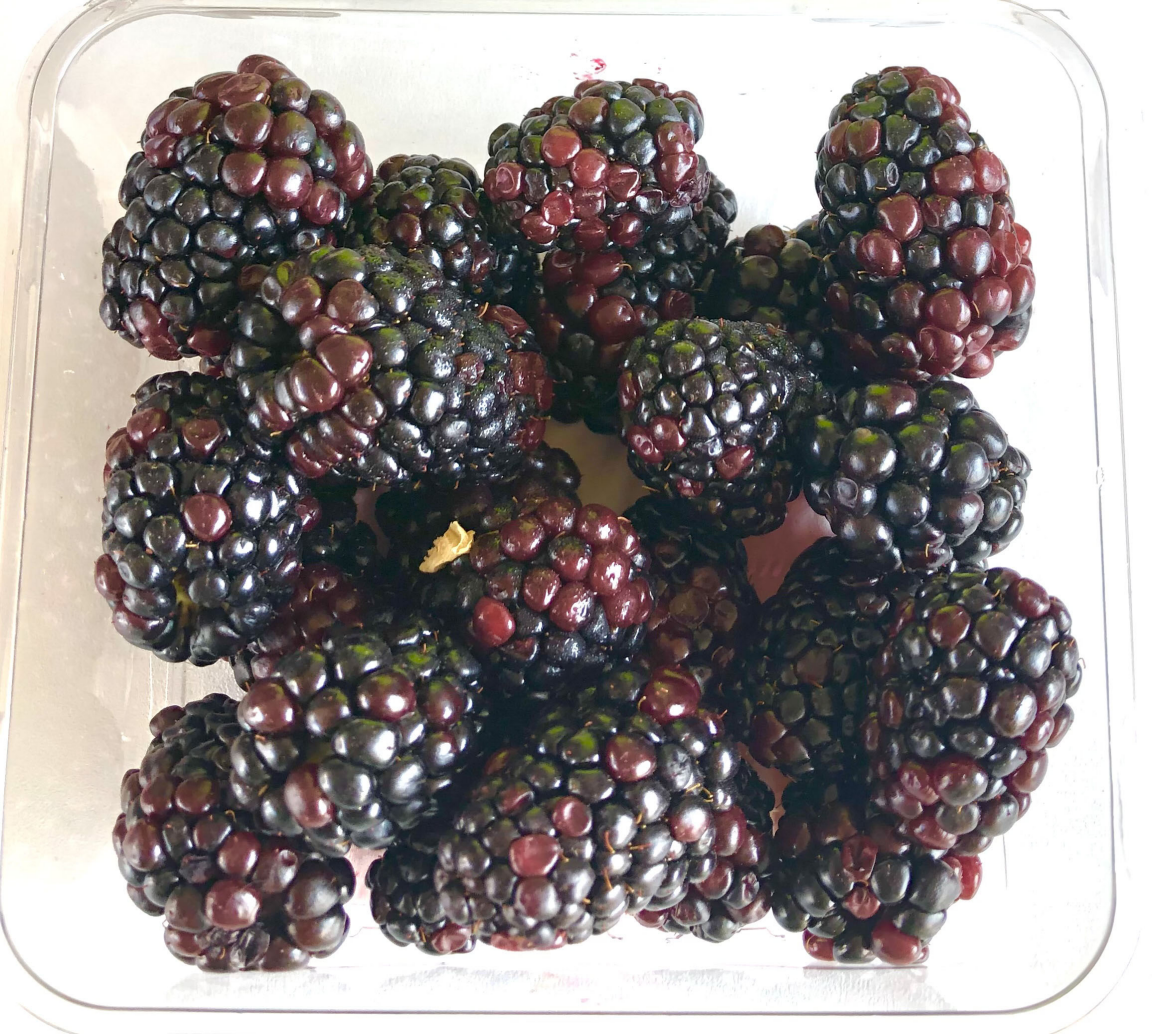
A clamshell of blackberry fruit with severe red drupelet reversion (RDR) in postharvest evaluations in the University of Arkansas System Division of Agriculture Fruit Breeding Program.

To evaluate the potential of ShinyFruit, we examined its ability to quantify red drupelet reversion (RDR), a specific postharvest disorder affecting the growing fresh-market blackberry (*Rubus* subgenus *Rubus*) industry (**Figure 1**). RDR refers to the phenomenon wherein fully black blackberries revert to a red color after shipping/storage, negatively impacting fruit quality and consumer perception (Clark and Finn, 2011). In an online survey of demographically diverse blackberry consumers, individuals strongly preferred images of blackberries with minimal RDR (Threlfall et al., 2020). These results were also validated in a subsequent in-person consumer sensory panel (Threlfall et al., 2021). In addition to deterring would-be consumers, severe RDR can incur immediate and apparent economic losses. According to USDA-AMS guidelines, entire lots of blackberries can be rejected if RDR damage affects at least 10% of the berry lot by volume or only 5% by volume if the damage is categorized as severe (United States Department of Agriculture, Agricultural Marketing Service, 2018). Much like other postharvest conditions, RDR is affected by genetic factors (Salgado and Clark, 2016; Lawrence and Melgar, 2018) and cultural practices such as temperature and handling at harvest (Edgley et al., 2019a; Armour et al., 2021), shipping vibration patterns (Pérez-Pérez et al., 2018), and nitrogen fertilizer application rates (Edgley et al., 2019b). On the cellular level, RDR is likely the result of mechanical cell disruption, separation, and loss of integrity in the upper mesocarp leading to the decompartmentalization and subsequent oxidative degradation of anthocyanin (phenolic) pigments (Edgley et al., 2019c; Kim et al., 2019).

Published methods for RDR detection are inconsistent or performed using subjective visual assessment. For instance, Clark and Perkins-Veazie (2011) evaluated RDR by categorizing entire berries as reverted or non-reverted based on a minimum threshold of three reverted drupelets, and Segantini et al. (2017) quantified RDR on individual berries by dividing the total number of reverted drupelets by total drupelets on each berry. Edgley et al. (2019a) devised a similar approach that also accounted for partially reverted drupelets. Developing a standardized, efficient, and objective technique would benefit breeders by providing the necessary framework for a scalable, simplified RDR screening protocol, improving their ability to select shipping tolerant genotypes.

Beyond color quality, morphological berry traits are often important to breeders as well. Large fruit size has been a key objective of the University of Arkansas (UA) System Division of Agriculture Fruit Breeding Program since its inception in 1964 (Clark, 1999). In fresh-market blackberry production, where fruit is harvested by hand, large fruit presents an obvious benefit to harvest efficiency. Surveyed consumers also tend to prefer blackberries that are large and oblong rather than small and round (Threlfall et al., 2020; Threlfall et al., 2021). Drastic gains in size have been achieved by modern cultivars like ‘Natchez’ (8.0 – 10.2 g/berry) (Clark and Moore, 2008), which can reach twice the size of the earliest UA releases (4.8 – 6.0 g/berry) (Moore et al., 1974). These large-fruited cultivars have approached maximum desirable size for packaging, but adequate size remains an important qualification for any new release. Like RDR, fruit size may easily be incorporated in an automated imaging pipeline, replacing the traditional use of scales or calipers.

With flexible, user-determined input settings, the utility of an image analysis pipeline may be extended to data collection in a wider array of morphological characteristics in blackberry and other fruits and vegetables, allowing breeders to have more versatility in selection methods. In the present study, we seek to compare the ShinyFruit software package, which has been designed with these specific objectives in mind, to ImageJ and more traditional phenotyping techniques in a blackberry population of diverse sizes and shipping qualities. We demonstrate one implementation of this tool in blackberry (*Rubus* subgenus *Rubus*) by comparing automated phenotypic measurements with those from traditional manual techniques.

## Materials and methods

### Plant material and harvest

Floricane blackberry fruit from fourteen UA System breeding selections and cultivars representing a diverse range of textures and susceptibility to RDR were harvested from 6 m plots located at UA System Fruit Research Station (FRS) in Clarksville, Arkansas in 2019, 2020, and 2021. The FRS site is located at 35°C 31’5”N and long. 93°C 24’12”W, in USDA hardiness zone 7b (United States Department of Agriculture, Agricultural Research Service, 2021), on Linker fine sandy loam. All plots evaluated were treated with standard production practices including and early spring application of ammonium nitrate (56 kg.ha^-1^ N) and a biweekly fertigation application of 20N-4.4P-17K from flowering to harvest. Liquid lime sulfur fungicide (94 L.ha^-1^) was applied during bud break, five weeks before first harvest, and three weeks before first harvest to minimize anthracnose (*Elsinoë veneta*), botrytis fruit rot (*Botrytis cinerea*), and cane and leaf rust (*Kuehneola uredines*). Multiple pesticides containing active ingredients zeta-cypermethrin, bifenthrin, and malathion were applied weekly from flowering until floricane harvest in June to control spotted wing drosophila (*Drosophila suzukii*). A bifenthrin-containing insecticide was also applied annually in October to control raspberry crown borer (*Pennisetia marginata*). All plants were trained to a four-wire, horizontal T-trellis with low and high wires at 0.5 m and 1.0 m height. Plants were tipped to 1.1 m height in mid-May and lateral branches were pruned in August. Plots were grown in black plastic mulch to reduce weed pressure.

The 14 genotypes evaluated included A-2444T, A-2453T, A-2454T, A-2491T, ‘Black Gem^™^’, ‘Black Magic^™^’, ‘Sweet-Ark^®^ Caddo’, ‘Natchez’, ‘Osage’, ‘Ouachita’, ‘Sweet-Ark^®^ Ponca’, ‘Prime-Ark^®^ Horizon’, ‘Prime-Ark^®^ Freedom’ and ‘Prime-Ark^®^ Traveler’. Blackberries were harvested at the shiny black stage in 500 mL clamshells on two separate harvest dates each year, with at least one week between harvest dates. All fruit was harvested after 10:00 AM, when temperatures were usually over 27°C, to encourage occurrence of red drupelet reversion (RDR) (Edgley et al., 2019a; Armour et al., 2021). Clamshells were filled just below the lid and placed directly into a portable cooler chilled by ice packs until they could be transported. In 2020 and 2021, harvested fruit samples were placed on a custom-built steel table for 30 minutes, with a vibrating surface that produced 2 mm of displacement and a frequency of 10 Hz. This treatment was intended to simulate shipping conditions that lead to RDR by replicating the findings of Pérez-Pérez et al. (2018). Samples were stored in an on-site refrigerator for seven days at 5°C and 90% relative humidity. Clamshells were removed from the refrigerator and allowed to reach room temperature before photographs were taken.

### Image capture

Photographs were collected seven days after harvest to allow RDR to occur during cold storage. Clamshells of fruit were photographed in a photo box (LimoStudio 16” x 16” Table Top Photo Photography Studio Lighting Light Tent Kit in a Box, AGG349; Las Vegas, NV) constructed on a countertop with a Canon EOS Rebel T3 (Tokyo, Japan) camera mounted directly above a green cutting board on which the fruit was staged. The camera was equipped with a Canon EFS 18-55mm lens (Tokyo, Japan) and images were captured in close-up mode with International Organization for Standardization (ISO) values ranging from 250-3200. Fruit from a single clamshell were divided into two portions to be photographed separately due to the size of the staging area. Number of berries photographed in each sample varied depending on berry size, with 10-15 berries included in larger genotypes and 20-25 included in smaller genotypes. In 2019, a standard US quarter dollar was included in each image as a size reference. In 2020 and 2021, an X-Rite ColorChecker Classic Mini (Grand Rapids, Michigan, USA) was included in each image and used as a size reference. The X-Rite ColorChecker was used to standardize the white balance in each image using the CIPF plugin in ImageJ, which is no longer publicly maintained. Unedited blackberry photographs used in this project that were taken in 2019, 2020, and 2021 are available at https://figshare.com/articles/figure/Blackberry_Images_2019/23859342, https://figshare.com/articles/figure/2020_blackberry_images/23859837, and https://figshare.com/articles/figure/Blackberry_images_2021/23860593.

### Fruit size

Length and width of five berries from each sample clamshell were measured using Pittsburgh digital calipers (Harbor Freight Tools, Camarillo, CA). Length of a fruit was defined as the distance between the abscission scar and the terminal drupelet. Berry width was defined as the maximum distance between drupelets on the equatorial plane. Fruit lengths and widths were only measured in 2020 and 2021.

### Subjective evaluation of red drupelet reversion

After all images were captured, each clamshell was subjectively evaluated on a ‘by-berry’ and ‘by-drupelet’ basis. In both methods, the Royal Horticultural Society Greyed-Purple 185-A color value (L*a*b = 34.4, 42.0, 12.7) was used as a reference threshold. Drupelets matching that value or brighter were counted as reverted. For the ‘by-berry’ method, the number of berries in each clamshell were recorded. Then, each fruit was individually inspected for reverted drupelets, with fruit having three or more red drupelets scored as reverted while fruit with two or fewer red drupelets were scored as not reverted following Clark and Perkins-Veazie (2011). For the ‘by-drupelet’ method, five berries from each clamshell were selected at random. Each fruit was mounted on a toothpick through the abscission scar to aid in viewing. Red drupelets, including fully red and any deviated from standard black toward red, were counted and marked with a paint pen. After red drupelet count, the remaining drupelets were counted in the same manner for a total drupelet count per fruit. Percent reverted drupelets were calculated for each of the five berries per clamshell following Segantini et al. (2017).

### ShinyFruit

**Figure 2:**
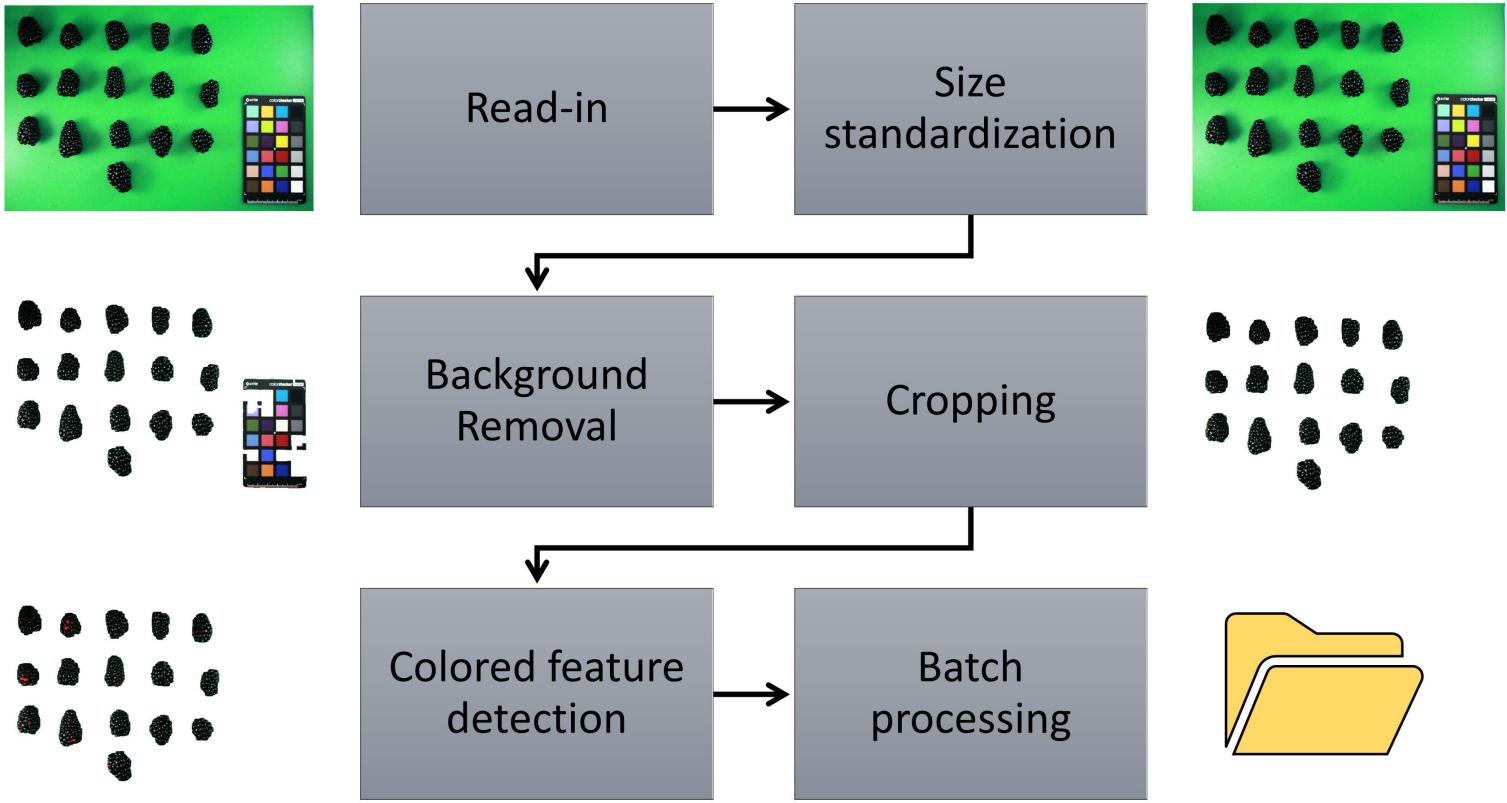
ShinyFruit image processing pipeline for colored feature detection using the example of red drupelet reversion (RDR) in blackberry fruit.

Source code for version 0.1.0 of the ShinyFruit software (Chizk, 2022) is maintained and publicly available on GitHub (https://github.com/mchizk1/ShinyFruit) under an MIT license. ShinyFruit’s image-processing utilities were built using the R packages magick (Ooms, 2021) and imager (Barthelme, 2022), which both offer efficient C++-based methods for image manipulation. The GUI was built using the shiny (Chang et al., 2022) package for web application development. Upon reading user-provided sample images in.jpg format, ShinyFruit automatically processes images in several ways to prepare for analysis and maximize efficiency. All images are downsized such that the maximum dimension does not exceed 1500 pixels. This reduces time required for batch-image processing at the potential expense of fine resolution. Contrast in images is increased by normalizing pixel values to span the full RGB range. Finally, differences in color intensity are sharpened, and the images are enhanced to reduce noisy or inconsistent pixel color values. Following read-in, the user may proceed through the image analysis pipeline detailed in **Figure 2**. Despeckling, which is implemented in the background removal and color feature detection steps, is achieved by successive shrinking and swelling of detected pixel groups. In this way, small, isolated groups of pixels (dust, juice, debris, etc.) are avoided during feature detection. Running the batch image analysis potentially generated two types of outputs including processed images and a comma separated value (csv) formatted text file containing requested data and implemented user settings for repeatability.

**Figure 3:**
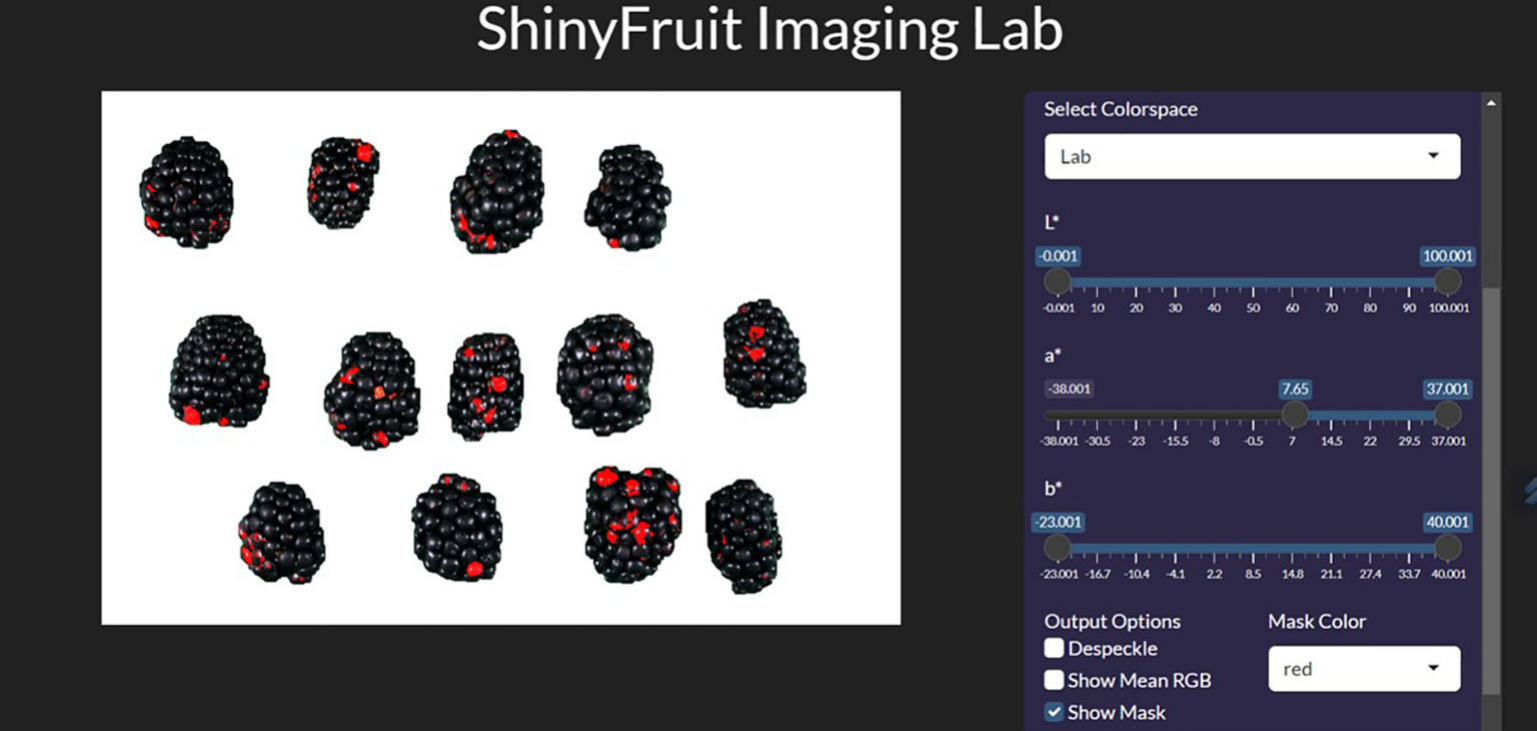
ShinyFruit general user interface (GUI) example during the colored feature detection step measuring blackberries.

For each year of this study, a single representative image of blackberry fruit containing observable levels of RDR was read into the ShinyFruit program to remove background pixels, calibrate size references and determine the appropriate color cutoff thresholds in the L*a*b* color space (**Table 1**). **Figure 3** provides an example of the ShinyFruit GUI at the color thresholding stage of image analysis. Size and color settings specific to each year of image data were uniformly applied to batch-process all images. In 2019, the diameter of a US quarter dollar included in each image was used as the known size reference. In 2020 and 2021, the ruler edge of the X-Rite ColorChecker Classic Mini was used as the known size reference. The location surrounding these size references was designated to be uniformly cropped out during image processing. Pixels with an a* value of greater than 7.51, 16.15, and 8.58 were counted as reverted in 2019, 2020, and 2021, respectively. No cutoff thresholds were needed for L* or b* values to identify red regions. Resulting images were output for visual post-analysis quality checking.

**Table 1:** ShinyFruit^i^ settings used for image processing and detection of blackberry red drupelet reversion (RDR) in 2019-2021.

Step	Year	L^i^	a	b
Background removal	2019	FR^ii^	>-10.00	FR
	2020	FR	>-12.00	FR
	2021	FR	>-8.50	FR
RDR detection	2019	FR	>7.51	FR
	2020	FR	>16.15	FR
	2021	FR	>8.58	FR

^i^Lab colorspace threshold values.

^ii^The full range (FR) of values were accepted for the associated colorspace channel.

### ImageJ

For image analysis, software version 1.53e (https://imagej.nih.gov/ij/download.html) was used with Java version 1.8.0_172. A custom-written ImageJ macro script maintained on GitHub (https://github.com/mchizk1/UA_Fruit_Breeding/tree/main/IJ_RDR) was used to perform image analysis in a two-step procedure that mimics the ShinyFruit workflow presented. One set of L*a*b* thresholds (0-255 scale) were used to remove pixels associated with the green background, and another set of L*a*b* thresholds were used to identify pixels associated with the reverted regions. The proportion of RDR was estimated as the number of reverted pixels divided by the total number of non-background (fruit) pixels. In an initial run, all images were passed through a consistent set of L*a*b* thresholds. All pixels with a* values greater than 110 were considered to be fruit, and pixels with a* values of greater than 138 were considered to be reverted. L* and b* thresholds were not used. Based on visual inspection of results, subsets of images were rerun under separate thresholds. The ImageJ script did not include any of the image pre-processing methods described in the ShinyFruit method, such as RGB normalization or color sharpening.

### Experimental design and statistics

All analyses were performed using R version 4.1.2 (R Core Team, 2022). An analysis of variance (ANOVA) was performed using type III sums of squares and following a randomized complete block design. Harvest date was used as the blocking effect, while genotype and year were treated as fixed and random effects respectively. Estimated marginal means were calculated using the emmeans package (Lenth, 2022), and Tukey’s honestly significant differences (HSD) were calculated using *P* < 0.05 for all dependent variables for which significant genotypic differences were detected. Pearson’s correlations were calculated for genotypic means across years and pairwise linear regression were fitted with the ggplot2 (Wickham, 2016) and ggpubr (Kassambara, 2020) packages to compare automated and manual data collection methods.

## Results

**Figure 4:**
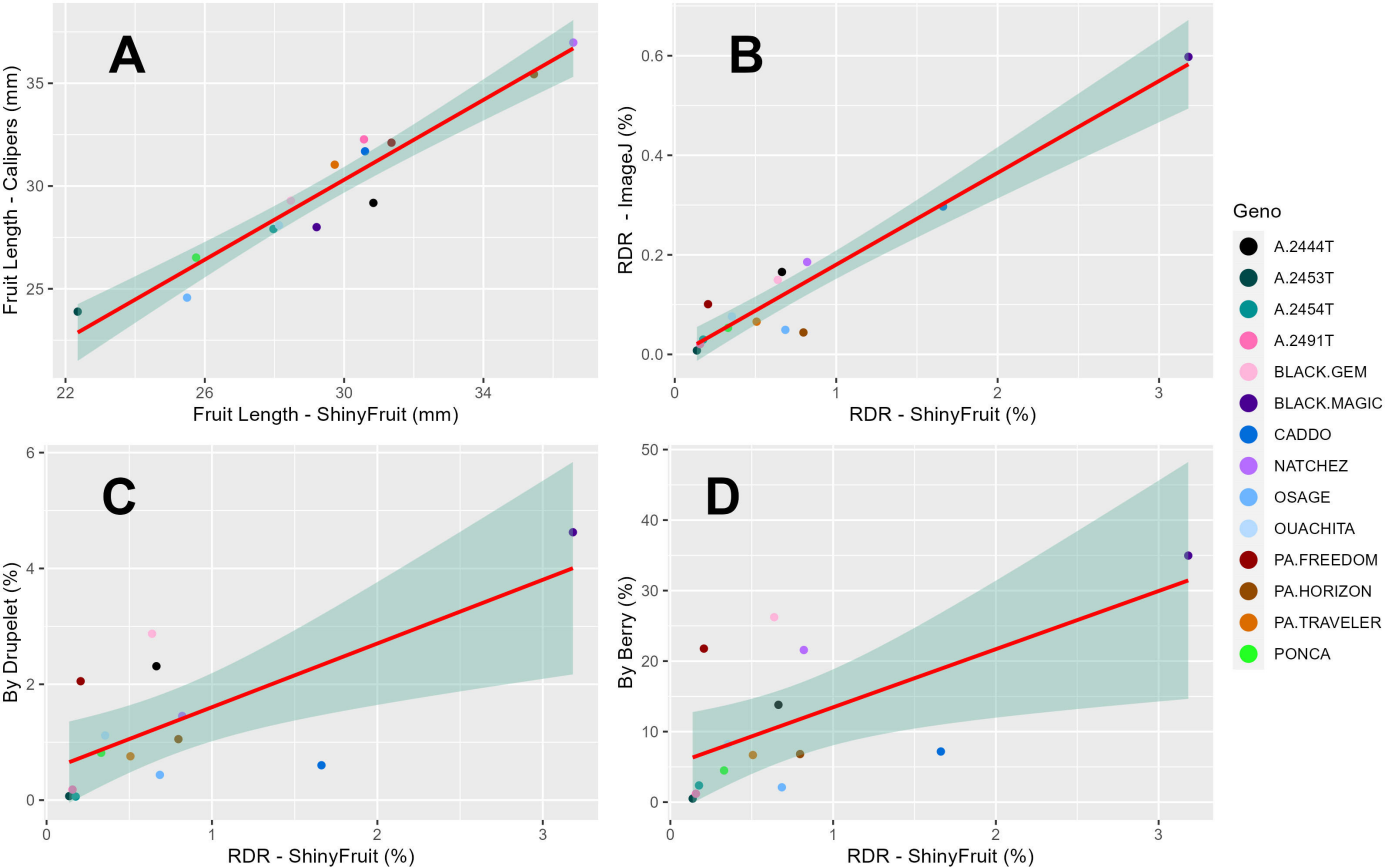
Regression of least square means across years (2019-2020) for 14 blackberry genotypes between ShinyFruit estimations and manual measurements of blackberry fruit length **(A)** and estimates of red drupelet reversion (RDR) from ImageJ **(B)** and ‘by-berry’ **(C)** and ‘by-drupelet’ **(D)** manual assessments.

ShinyFruit, manual counting ‘by-berry’, and manual counting ‘by-drupelet’ all detected genotypic differences for fruit length, but none of these methods detected genotypic differences in fruit width. Genotype by year interactions for fruit length and fruit width were only significant in the ShinyFruit analysis (**Table 2**). ‘Prime-Ark^®^ Freedom’ and ‘Natchez’ were both shorter in 2020 than in 2021, and this difference was most apparent in the ShinyFruit dataset. Across years, ‘Natchez’ produced the longest fruit regardless of method, but the caliper-based method only distinguished ‘Natchez’ significantly from ‘Osage’ and A-2453T (**Table 2**). The latter two genotypes consistently produced the shortest fruit, regardless of method. Genotypic differences between mean fruit lengths were more pronounced in the ShinyFruit analysis. According to the ShinyFruit results, ‘Natchez’ and ‘Prime-Ark^®^ Horizon’ had significantly longer fruit than all other genotypes except for ‘Prime-Ark^®^ Freedom’. Similarly, A-2453T fruit was significantly shorter than all other genotypes except for ‘Osage’ and ‘Sweet-Ark^®^ Ponca’. ShinyFruit and caliper-based methods for measuring fruit length were very tightly correlated (**Table 3**; **Figure 4A**, *r* = 0.962).

**Table 2:** Least square means of red drupelet reversion and fruit size as measured by image-based estimation and manual methods (2019-2021^i^).

Geno	Red drupelet reversion (%)								Fruit Length (mm)^i^				Fruit Width (mm)			
	ShinyFruit^iv^		ImageJ^vi^		By-drupelet^vii^		By-berry^viii^		ShinyFruit		Calipers		ShinyFruit		Calipers	
A-2444T	0.66	a^v^	0.17	ab	2.31	ab	13.80	ab	30.84	b	29.18	ab	24.05		23.98	
A-2453T	0.14	a	0.01	a	0.07	a	0.50	a	22.35	a	23.89	a	19.90		21.66	
A-2454T	0.18	a	0.03	a	0.06	a	2.37	a	27.97	b	27.91	ab	23.48		23.90	
A-2491T	0.16	a	0.02	a	0.18	a	1.19	a	30.57	b	32.27	b	21.16		21.58	
Black Gem	0.64	a	0.15	ab	2.87	ab	26.23	ab	28.47	b	29.29	ab	21.92		23.24	
Black Magic	3.18	b	0.60	b	4.62	b	34.97	b	29.21	b	28.00	ab	22.67		22.05	
Natchez	0.82	a	0.19	ab	1.45	a	21.57	ab	36.58	c	36.98	b	23.74		23.56	
Osage	0.69	a	0.05	a	0.44	a	2.10	a	25.49	ab	24.57	a	22.50		22.59	
Ouachita	0.35	a	0.08	a	1.12	a	8.23	a	28.13	b	28.09	ab	23.92		23.74	
Prime-Ark^®^ Freedom	0.21	a	0.10	ab	2.05	ab	21.77	ab	31.36	bc	32.11	b	24.31		25.04	
Prime-Ark^®^ Horizon	0.80	a	0.04	a	1.05	a	6.81	a	35.45	c	35.44	b	21.77		22.71	
Prime-Ark^®^ Traveler	0.51	a	0.07	a	0.76	a	6.68	a	29.73	b	31.04	b	21.00		21.87	
Sweet-Ark^®^ Caddo	1.66	ab	0.30	ab	0.60	a	7.18	a	30.60	b	31.69	b	22.37		22.82	
Sweet-Ark^®^ Ponca	0.33	a	0.05	a	0.82	a	4.49	a	25.75	ab	26.52	ab	21.06		20.73	
*P_G_^ii^*	0.002		0.002		0.002		0.001		0.014		0.001		0.504		0.427	
*P_GY_^iii^*	0.767		0.576		0.374		0.666		0.021		0.825		0.044		0.769	

^i^Fruit length and width were only measured in 2020 and 2021.

^ii^P values for genotypes.

^iii^P values for genotype by year interactions.

^iv^Open-source software available at https://github.com/mchizk1/ShinyFruit.

^v^Letters indicate significant differences between genotypes (P < 0.05) using Tukey's Honestly Significant Difference.

^vi^Open-source software available at https://imagej.nih.gov/ij/download.html.

^vii^The mean percentage of reverted drupelets per blackberry.

^viii^The mean percentage of berries in a clamshell that had three or more reverted drupelets.

**Table 3:** Pearson correlation of genotypic mean fruit characteristics between automated and manual measurements of red drupelet reversion (RDR) and fruit length in blackberry.

		RDR						Fruit length			
		ImageJ		By-drupelet		By-berry		ShinyFruit		Calipers	
RDR	ShinyFruit^i^	0.958	**	0.696	**	0.621	*	0.179	^NS^	0.058	^NS^
	ImageJ^ii^			0.795	**	0.756	**	0.197	^NS^	0.081	^NS^
	By-drupelet^iii^					0.940	**	0.225	^NS^	0.085	^NS^
	By-berry^iv^							0.361	^NS^	0.265	^NS^
Fruit Length	ShinyFruit									0.962	**

NS, *, and ** Nonsignificant or significant at P < 0.05 or 0.01, respectively.

i Open-source software available at https://github.com/mchizk1/ShinyFruit.

^ii^Open-source software available at https://imagej.nih.gov/ij/download.html.

^iii^The percentage of reverted drupelets per blackberry.

^iv^The percentage of berries in a clamshell that had three or more reverted drupelets.

ShinyFruit, ImageJ, manual counting ‘by-berry’, and manual counting ‘by-drupelet’ were all capable of distinguishing differences between genotypes across all three years for RDR (**Table 2**). ‘Black Magic™’ had significantly more reversion than most other genotypes tested (**Table 2**), regardless of phenotyping method. Neither of the manual RDR counting techniques identified differences in RDR between ‘Sweet-Ark^®^ Caddo’ and lower-reversion genotypes, but both ShinyFruit and ImageJ did. ShinyFruit and ImageJ based estimates of RDR were highly correlated (**Table 3**; **Figure 4B**, *r* = 0.958). The two manual RDR counting methods were much more highly correlated with one another (**Table 3**, *r* = 0.940) than with ShinyFruit or ImageJ RDR estimates, but both were still significantly correlated with image-based results. ImageJ was more tightly correlated with manual counting techniques than ShinyFruit (**Table 3**), but ShinyFruit RDR estimates were consistently higher than those from ImageJ (**Table 2**). Drupelet-based RDR estimation was more tightly correlated with both image-based methods (**Table 3**; **Figure 4C**, *r* = 0.696-0.795), although ‘by-berry’ RDR estimation was significantly correlated as well (**Table 3**; **Figure 4D**, *r* = 0.621-0.756). The image-based ImageJ method and manual ‘by-drupelet’ and ‘by-berry’ RDR methods all grouped ‘Black Magic™’, ‘Black Gem™’, ‘Prime-Ark^®^ Freedom’, and A-2444T together in the highest reversion group. The ShinyFruit RDR method also grouped ‘Black Magic™’ among the highest RDR genotypes, but underestimated RDR in ‘Black Gem™’, ‘Prime-Ark^®^ Freedom’, and A-2444T. ‘Sweet-Ark^®^ Caddo’ only grouped among the highest RDR genotypes using the ShinyFruit estimation method.

## Discussion

Manual measures of RDR using the ‘by-drupelet’ and ‘by-berry’ method were generally low compared to previous studies that have implemented similar methods. Segantini et al. (2017) and Felts et al., (2020) observed RDR ranges of 0.7-6.1% and 2.43-8.06%, respectively, using the ‘by-drupelet’ method to evaluate germplasm closely related to the materials included in this study. In contrast, our ‘by-drupelet’ RDR observations only ranged from 0.06-4.63%. Similarly, Armour et al. (2021) observed a range of 1.42-79.83% using the by-berry RDR estimation method in closely related germplasm, while our own observations ranged from 0.50-34.97%. Each of these studies considered only partially overlapping samples of UA germplasm, which could partially account for differences in observed RDR ranges. However, this comparison suggests that our vibration treatment following Pérez-Pérez et al. (2018) was likely insufficient in promoting higher levels of RDR expression across the population. There were several notable similarities between our observed genotypic rankings for RDR expression levels and those reported by others. Unsurprisingly, we observed that ‘Black Magic™’ consistently grouped with the highest RDR genotypes using manual techniques. These findings are consistent with Armour et al. (2021), who reported that ‘Black Magic™’ had the softest fruit and highest RDR among the seven genotypes evaluated in that study. ‘Natchez’ also had moderate to high levels of RDR in previous studies (Armour et al., 2021; Felts et al., 2020), although ‘Natchez’ was only in the highest RDR statistical group using the ‘by-berry’ RDR method in this study (**Table 2**). This discrepancy could suggest a bias present in the by-berry method, which may overestimate RDR in large-fruited genotypes like ‘Natchez’ with many drupelets (**Table 2**). This bias was not confirmed by any statistically significant correlation between fruit length and ‘by-berry’ RDR estimation, but of all the RDR methods, the ‘by-berry’ method was most correlated with fruit length (**Table 3**). As noted in previous studies of RDR, A-2453T, ‘Osage’, and ‘Prime-Ark^®^ Traveler’ all consistently grouped with the least reverted genotypes (Armour et al., 2021; Felts et al., 2020). Among these low-RDR genotypes, A-2453T and ‘Prime-Ark^®^ Traveler’ are both noteworthy for their firm texture (Armour et al., 2021) and shipping potential (Clark and Salgado, 2016; Salgado and Clark, 2016).

ShinyFruit rankings of RDR intensity were similar to manual methods and ImageJ in most respects, with a few exceptions in the intermediate ranges. According to both ShinyFruit and ImageJ, ‘Sweet-Ark^®^ Caddo’ grouped with ‘Black Magic™’ in the highest RDR group. ImageJ and both manual techniques also grouped A-2444T, ‘Black Gem™’, and ‘Prime-Ark^®^ Freedom’ together with ‘Black Magic™’ as the genotypes with highest RDR. ShinyFruit RDR estimates were more highly correlated with the manual ‘by-drupelet’ (*r* = 0.696) method than the ‘by-berry’ method (*r* = 0.621). This was consistent with ImageJ correlations and aligns with expectations, since the ‘by-berry’ method is expected to be biased by fruit size and the other two methods are not. Even so, the manual ‘by-berry’ and ‘by-drupelet’ methods were much more correlated with one another (*r* = 0.904) than either was with ShinyFruit or ImageJ (**Figure 4**). This may partly be explained by the fact that both manual estimates considered all drupelets on each berry, and each drupelet was categorically considered to be either reverted or non-reverted. Unlike the manual techniques, ShinyFruit and ImageJ estimates only considered the upper surface area of berry samples that were visible in each image. Furthermore, instead of categorizing each drupelet as reverted or non-reverted, ShinyFruit and ImageJ categorize individual pixels based on color thresholds. Thus, image-based estimates can provide RDR estimates that accurately account for partial reversion of drupelets. Similarly, by using two separate reversion thresholds, one could measure reversion with varying degrees of color intensity as suggested by Edgley et al. (2019c). Future implementations of ShinyFruit should be able to compensate for the problem of RDR half-estimation, from only measuring one side of the fruit, by doubling the amount of fruit imaged. Through manual inspection of ShinyFruit output images, it is also clear that the digital image analysis pipeline also detected certain non-RDR discolorations of features such as desiccated, ruptured, or anthracnose-infected drupelets. RDR on berries with excessive glossiness may have also been underestimated with ShinyFruit, since the reflection of light can mask the color of the drupelets underneath. Future work may investigate this hypothesis through a correlation of ShinyFruit RDR estimation and glossiness. If such a relationship exists, future pipelines may consider glossiness as a covariate for ShinyFruit RDR estimation.

Two key differences were observed between the image-based RDR detection methods tested. Of the two image-based RDR phenotyping methods tested, ImageJ held the tightest correlations with manual by-drupelet (*r* = 0.795) and by-berry methods (*r* = 0.756), but ShinyFruit appears to be far more sensitive to detecting low-levels of reversion. Even in the high-reversion ‘Black Magic™’, ImageJ only estimated a mean RDR of 0.60% (**Table 2**), while ShinyFruit estimated 3.18% for the same genotype. This increased sensitivity is probably attributable to the image preprocessing capabilities contained in the ShinyFruit package, which ease the process of color-based thresholding while minimizing noisy data. Just as with the manual techniques, both image-based techniques were very highly correlated with one another (**Table 3**, *r* = 0.958). However, the slight differences in correlation with manual techniques are more difficult to interpret. While it is possible that ShinyFruit is less accurate than other methods, James et al. (2022) points out that automated methods approach the problem of color quantification in a fundamentally different way than manual techniques. Although automated methods do not eliminate subjectivity entirely, the user-determined color thresholds are evenly applied, producing greater consistency in results. For this reason, the tightness of correlations between automated and manual methods should not be intensely scrutinized. Instead, it may be more beneficial to focus on sensitivity of detection and ease-of-use. In contrast with the ImageJ macro method, ShinyFruit presents strong advantages in these areas since it requires no coding expertise to use. Thus, ShinyFruit may present an attractive alternative in crops like strawberry, where color quantification is important, but existing solutions require some coding capabilities (Zingaretti et al., 2021) or specialized hardware (Liming and Yanchao, 2010).

ShinyFruit measurements of fruit length and RDR were tightly correlated with those from manual data collection techniques. This resemblance is especially clear for fruit length measurements, which were within 1 mm of caliper-based measurements in all genotypes except for A-2453T and ‘Black Magic™’ (**Table 2**). Occasional and slight differences between fruit length measurements could arise from a slight difference in the way ShinyFruit estimates length compared to calipers. ShinyFruit considers the length between the uppermost detected berry pixel from the lowest berry pixel. Thus, berry orientation is key in producing accurate results. Calipers measure the length between the peduncle attachment point and the terminal drupelet. In addition, ShinyFruit relies on user-provided size standardization from a single sample image, and it assumes that the fixed camera height is kept consistent between other images analyzed in the same batch. Violation of this assumption could result in inaccuracies. Despite very tight correlations between length phenotyping methods (**Table 3**, *r* = 0.962), Genotype by year interactions were also present in ShinyFruit-based fruit length estimations, but not in caliper-based measurements (**Table 2**). This could stem from slight differences in camera settings, ambient lighting, or ShinyFruit parameters between years, which may affect genotypic length measurements differently based on interfering qualities such as glossiness or turgidity. It seems more likely, based on a comparison of means *P* values within years, that this interaction could indicate a ‘real’ effect which is only detectable in larger sample sizes (up to 25 per image). ‘Prime-Ark^®^ Freedom’ provides a clear example of this interaction. Both ShinyFruit and caliper methods indicate that mean fruit lengths of ‘Prime-Ark^®^ Freedom’ were at least 5 mm longer in 2021 than in 2020, but only ShinyFruit statistically distinguished this genotype from the shortest genotypes in 2021. ShinyFruit may provide an improvement in accuracy, even compared to direct caliper measurement of fruit length, because of its enhanced throughput. In the present study, only five randomly sampled berries were measured with calipers, while entire clamshells were easily analyzed using ShinyFruit. The ability to measure greater numbers of berries reduces experimental error and improves the ability of the researcher to make strong inferences between genotypes or treatment groups.

Based on the evidence presented, ShinyFruit appears to be capable of sufficiently estimating RDR and fruit size in blackberry. In fact, ShinyFruit was recently used in genome-wide association analysis of RDR across 300 fresh-market blackberry genotypes evaluated over three years (Chizk et al., 2023). But fruit size and RDR are only two of many potential applications for this tool. In blackberry alone, protocols could be developed to mimic existing manual phenotyping methods for glossiness (Segantini et al., 2017) or white drupelet disorder (Stafne et al., 2017). Moreover, ShinyFruit’s potential may extend beyond blackberries, as it can be adapted and applied to various niches in other crop species, contributing to advancements in high-throughput image analysis. The tool’s algorithms and user-friendly interface make it accessible for researchers working with different horticultural crops, enabling them to leverage its capabilities for diverse phenotyping tasks. Efficient strategies for estimating fruit size could be applied to numerous horticultural products by imitating ImageJ-based strategies (Cortes et al., 2017; Manolikaki et al., 2022), but the detection of color-based features could provide an even greater number of implementations. ShinyFruit could be used to quantify and characterize descriptive color value distributions for specific cultivars by using the optional ‘Color Profile’ feature. This feature reports the nearest matched RHS color descriptor to maximum, minimum, and median RGB color values in detected features. Such information may be of value in providing a standardized color description for new releases with unique color characteristics. Diseased or necrotic regions of fruit or leaves could easily be quantified by ShinyFruit following the approach used by Stewart and McDonald (2014) or Maloney et al. (2014) in wheat. ShinyFruit is an open-source response to proprietary image analysis tools, like Assess, and it is much simpler to use than ImageJ.

James et al. (2022) highlight the need for automated image analysis tools in strawberry trait automation, emphasizing the potential benefits of high-throughput phenotyping strategies. Fruit quality attributes have already been assessed using image-based techniques in many fruit species, including apple (Throop et al., 2005), citrus (Blasco et al., 2007), mango (Kang et al., 2008), and banana (Mendoza and Aguilera, 2006). In each of these situations, ShinyFruit could be employed as a GUI-based alternative that assumes no prior skillsets or specialized hardware. More modern methods have been used in grapes (Sozzi et al., 2022) and leverage machine learning algorithms to offer an even higher throughput solution, but these methods also come with certain drawbacks that are worth considering. Currently they are highly effective for simple tasks, like object detection, but they require training data that are representative of test image sets, and these machine learning algorithms are not easily customizable to changing thresholds or alternate traits. Future updates to ShinyFruit will focus on developing image segmentation algorithms for counting aggregated features, such as blackberry drupelets or grapes on a cluster. With guidance from the expert user, and no necessary training data, ShinyFruit should reduce barriers to high throughput phenotyping in a wide range of traits and species.

## Conclusion

Using the ShinyFruit R package (a flexible GUI-enabled tool for estimating size and color attributes in horticultural products), we observed tight correlations with manual measurements of blackberry fruit length (r = 0.96) and moderate correlations with manual measurements of RDR using the ‘by-drupelet’ (r = 0.70) and ‘by-berry’ (r = 0.62) methods. Compared to ImageJ, ShinyFruit was more sensitive to the low levels of RDR observed, but was slightly less correlated with manual methods. ShinyFruit RDR values aligned with manual measurements on high RDR and low RDR genotypes, but intermediate rankings between methods shifted slightly. Unlike the ‘by-berry’ method, ShinyFruit RDR phenotyping is unbiased by fruit size, but additional fruit should be harvested to account for ShinyFruit’s implicit half-measurement problem. Strategies should be developed in future studies to implement ShinyFruit phenotyping in fruit morphology and color-based trait measurement across a wide range of species and horticultural products.

## Data availability statement

The original contributions presented in the study are publicly available. Unedited blackberry photographs used in this project that were taken in 2019, 2020, and 2021 are available at https://figshare.com/articles/figure/Blackberry_Images_2019/23859342, https://figshare.com/articles/figure/2020_blackberry_images/23859837, and https://figshare.com/articles/figure/Blackberry_images_2021/23860593. Source code and documentation for version 0.1.0 of the ShinyFruit software is maintained and publicly available on GitHub (https://github.com/mchizk1/ShinyFruit).

## Author contributions

TC designed the ShinyFruit app, phenotyped blackberry samples, analyzed data, and drafted the manuscript in collaboration with MW. MW wrote the grant proposals that funded this research, supervised TC as a doctoral student, and provided overall conceptual guidance. JL oversaw management of the plants used in this study and JC developed all the varieties and breeding selections used in the trial. All authors contributed to the article and approved the submitted version.
